# Prevalence of obstructive coronary artery disease in asymptomatic cancer patients with elevated troponin levels

**DOI:** 10.1186/s40959-025-00432-4

**Published:** 2026-01-10

**Authors:** Jannek Brauer, Sebastian W. Romann, Christian Stengele, Lukas F. Entenmann, Daniel Finke, Hugo A. Katus, Evangelos Giannitsis, Norbert Frey, Lorenz H. Lehmann

**Affiliations:** 1 Department of Internal Medicine III: Cardiology, Angiology & Pulmonology, Cardio-Oncology unit, Heidelberg University Hospital, Im Neuenheimer Feld 410, Heidelberg, 69120 Germany; 2 DZHK (German Centre for Cardiovascular Research), Partner Site Heidelberg/Mannheim, Heidelberg, 69120 Germany; 3 Lithuanian University of Health Sciences, Kaunas, 44499 Lithuania; 4 German Cancer Research Center (DKFZ), Heidelberg, 69120 Germany; 5 Clinical Cancer Registry, National Centre for Tumour Diseases (NCT) Heidelberg, Heidelberg, 69120 Germany

**Keywords:** Cardio-oncology, Coronary arterial disease, Cardiac biomarkers, Coronary heart disease, Cardiotoxicity

## Abstract

**Background:**

High-sensitivity cardiac troponin T (hs-cTnT) is frequently elevated in cancer patients, but its clinical implications remain uncertain. We aimed to determine the prevalence of obstructive coronary artery disease (CAD) as a cause of hs-cTnT elevation and to evaluate the impact of cardiovascular risk factors and oncologic therapies.

**Methods:**

Between 2016 and 2023, 810 consecutive cancer patients with elevated hs-cTnT values presenting to the cardio-oncology outpatient clinic in Heidelberg were included. All patients underwent standardized cardiovascular assessment. Further imaging or invasive evaluation was performed in cases of unexplained biomarker elevation. Logistic regression was applied to identify predictors of CAD. Oncologic therapies were categorized according to ESC cardio-oncology guidelines into low, moderate, or high cardiotoxic risk.

**Results:**

Overall, 340/810 (42.0%) patients revealed alternative explanations for hs-cTnT elevation (e.g., atrial fibrillation, pulmonary embolism, and renal dysfunction). Among the remaining 470/810 (58.0%) patients evaluated for suspected CAD, 47/470 (10.0%) underwent percutaneous coronary intervention for obstructive CAD, whereas 423/470 (90.0%) showed no indication for revascularization. In multivariable analyses, younger age, female sex, preserved left ventricular ejection fraction, and normal NT-proBNP levels were independently associated with the absence of obstructive CAD. Among the 423 patients without obstructive CAD, 286/423 (67.6%) received active oncologic therapy, and 78.3% were exposed to moderate- or high-risk cardiotoxic agents.

**Conclusions:**

In cancer patients with elevated hs-cTnT, obstructive CAD is present in only a minority of cases. Traditional cardiovascular risk factors predict CAD, but therapy- and disease-related cardiac injury appear to be major contributors, highlighting the need for refined risk stratification.

## Introduction

Cardiovascular disease is a leading cause of morbidity and mortality in patients with cancer, and its coexistence with malignancy has become a central focus of cardio-oncology [1–4]. Coronary artery disease (CAD) is of particular concern because it may not only influence cancer treatment strategies but also adversely affect long-term prognosis [5, 6]. Cancer patients represent a particularly vulnerable population owing to shared cardiovascular and cancer risk factors, including but not limited to cigarette smoking, diabetes and hypertension. Both the malignancy itself and multimodal therapies (e.g., chemotherapy, radiotherapy, and surgery) disrupt cardiovascular homeostasis and contribute to CAD development [7–9]. Additionally, traditional cardiovascular risk factors, such as obesity, smoking, hypertension, and diabetes mellitus, which are also implicated in carcinogenesis, further exacerbate the bidirectional relationship between malignancy and cardiovascular disease [10, 11].

Despite the increasing recognition of this clinical overlap, the role of cardiac biomarkers in detecting or predicting CAD or other reasons within the oncologic population remains insufficiently explored [12, 13]. Notably, a considerable proportion of patients with cancer exhibit elevations in high-sensitivity cardiac troponin T (hs-cTnT) in the absence of angiographically documented obstructive CAD, underscoring the need to delineate alternative mechanisms of myocardial injury and their clinical implications [14–16]. Cancer therapies themselves represent a major contributor to myocardial injury. Anthracyclines, HER2-targeted agents, immune checkpoint inhibitors, and thoracic radiotherapy have all been implicated in direct cardiotoxic effects, leading to structural, functional, and biomarker-detected evidence of myocardial damage [15]. Importantly, these therapy-related mechanisms may coexist with traditional cardiovascular risk factors, complicating the attribution of biomarker elevation to CAD versus treatment-induced cardiotoxicity. Beyond ischemic and non-ischemic myocardial injury, mild elevations in hs-cTnT may also reflect increased myocardial workload or physiological stress [17]. In cancer patients, conditions such as anaemia, fever, tachyarrhythmias, or chronic inflammation may similarly increase cardiac workload and contribute to low-grade troponin elevation [15]. However, whether such hs-cTnT elevations predominantly indicate obstructive CAD or instead reflect nonischaemic mechanisms of myocardial injury related to cancer therapies or comorbid conditions remains unknown.

In this study, we aimed to determine whether elevated cardiac biomarkers in patients with cancer reliably indicate the presence of obstructive CAD or whether they more often reflect alternative, nonischaemic mechanisms of myocardial injury.

## Methods

### Patients

Between 2016 and 2023, a total of 1,035 oncologic patients with at least one elevated high-sensitivity cardiac troponin T (hs-cTnT) value during follow-up were identified at the cardio-oncology outpatient clinic of the National Center for Tumor Diseases (NCT), Heidelberg, Germany. For all analyses, we used the index elevated hs-cTnT measurement obtained at the cardio-oncology outpatient clinic. The timing of this index measurement was categorized as before, during, or after oncological therapy based on the documented date of the most recent treatment exposure. Of these 1035 oncologic patients, 225 patients were excluded because of missing data, leaving 810 patients who were included in the final analysis.

The cardio-oncology outpatient clinic is embedded within the standard-of-care framework and offers standardized cardiovascular evaluation for unselected cancer patients, irrespective of cancer type, stage, or treatment regimen. The clinic serves as an interdisciplinary interface between oncology and cardiology and provides structured cardiovascular monitoring throughout the oncologic care continuum [18]. Admission is based on the considerations of the treating oncologists. Prospective patient collection was based on a study protocol approved by the Ethics Committee of the Medical Faculty, University Heidelberg (S-286/2017, 390/2011).

All patients underwent a standardized cardiovascular evaluation at baseline, which included a comprehensive medical history, physical examination, 12-lead electrocardiography (ECG), and two-dimensional transthoracic echocardiography (2D-TTE). In addition, the serum levels of cardiac biomarkers (high-sensitivity cardiac troponin T (hs-cTnT) and N-terminal pro–B-type natriuretic peptide (NT-proBNP)) were assessed. In the case of an unexplained increase in hs-cTnT, defined as the absence of atrial fibrillation, LVEF < 50%, chronic kidney disease stage KDIGO > 2 (glomerular filtration rate (eGFR) < 60 mL/min/1.73 m²) or pulmonary embolism, further diagnostics were initiated to rule out the presence of obstructive CAD.

The primary endpoint was the diagnosis of coronary artery disease (CAD), confirmed by either invasive (e.g., coronary angiography) or non-invasive anatomical or functional imaging, i.e., coronary computed tomography angiography (CTA), cardiac magnetic resonance imaging (MRI) or stress echocardiography. Relevant obstructive CAD was defined as a luminal obstruction of 50% or more of at least one major epicardial coronary artery or major branch. A history of CAD also included patients with prior PCI. PCI was performed in the presence of ischaemic symptoms, evidence of inducible myocardial ischaemia, and/or a relevant finding on intraprocedural FFR testing. Further diagnostic cardiac work-up, including CTA, MRI, or invasive coronary angiography, was performed on the basis of the clinical presentation, echocardiographic findings, and individual cardiovascular risk profile, in accordance with the most recent guidelines for the diagnosis and management of acute and chronic coronary syndromes and heart failure [19–21]. The decision whether to perform CTA or stress testing in case of suspected coronary artery disease, was made by a cardiologist. In general, younger patients (absence of calcification, < 65 years) received CT-scans, while older patients and patients with the history of CAD more frequently received stress-tests.

### Data acquisition

Patient-specific data were prospectively collected and supplemented by retrospective data extraction from electronic medical records via the Cardiac Research Data Warehouse (RWH). The collected information included 12-lead electrocardiograms (ECGs), laboratory results, transthoracic echocardiographic measurements, cardiac MRI/CT findings, and invasive coronary angiography reports.

High-sensitivity cardiac troponin T (hs-cTnT) levels were measured in plasma samples via the Elecsys^®^ Troponin T high-sensitivity assay (Roche Diagnostics, Mannheim, Germany) on a Cobas^®^ e602 immunoassay analyser at the central laboratory of Heidelberg University Hospital. On the Cobas e602 platform, the analytical characteristics of the hs-cTnT assay were as follows: limit of blank (LoB), 3 ng/L; limit of detection (LoD), 5 ng/L; 10% coefficient of variation (CV), 13 ng/L; and 99th percentile upper reference limit, 14 ng/L [22, 23]. N-terminal pro–B-type natriuretic peptide (NT-proBNP) concentrations were determined via the Stratus^®^ CS Acute Care™ NT-proBNP assay (Siemens AG, Berlin and Munich, Germany).

Transthoracic echocardiography was performed via a Vivid E9 ultrasound system (General Electric, Milwaukee, WI, USA). Image acquisition was ECG-triggered, with a minimum of three cardiac cycles per image. The left ventricular ejection fraction (LVEF) was assessed by an experienced cardiologist via the biplane Simpson’s method in the apical 2- and 4-chamber views.

### Statistical analysis

All the data were collected and processed in R (version 4.0.5; R Foundation for Statistical Computing, Vienna, Austria) via custom scripts. Categorical variables were compared via the chi-square test or Fisher’s exact test, as appropriate. Continuous variables were analysed via the unpaired Student’s t test or the Mann–Whitney U test in the case of nonnormally distributed data. A p value < 0.05 was considered to indicate statistical significance.

A multivariable logistic regression model was used to identify predictors of the absence of obstructive CAD. The results are presented as odds ratios (ORs) with 95% confidence intervals (CIs).

For data visualization, the ggplot2 (version 3.3.2), ggpubr (version 0.4.0), and survminer (version 0.4.7) packages were employed. Flow charts were created via the DiagrammeR package (version 1.0.11), and Sankey diagrams were generated via the networkD3 package (version 0.4.1) to illustrate oncologic treatment patterns. All the tables were prepared via the flextable package (version 0.9.10).

## Results

### Diagnostic evaluation and management of elevated hs-cTnT

**Fig. 1:**
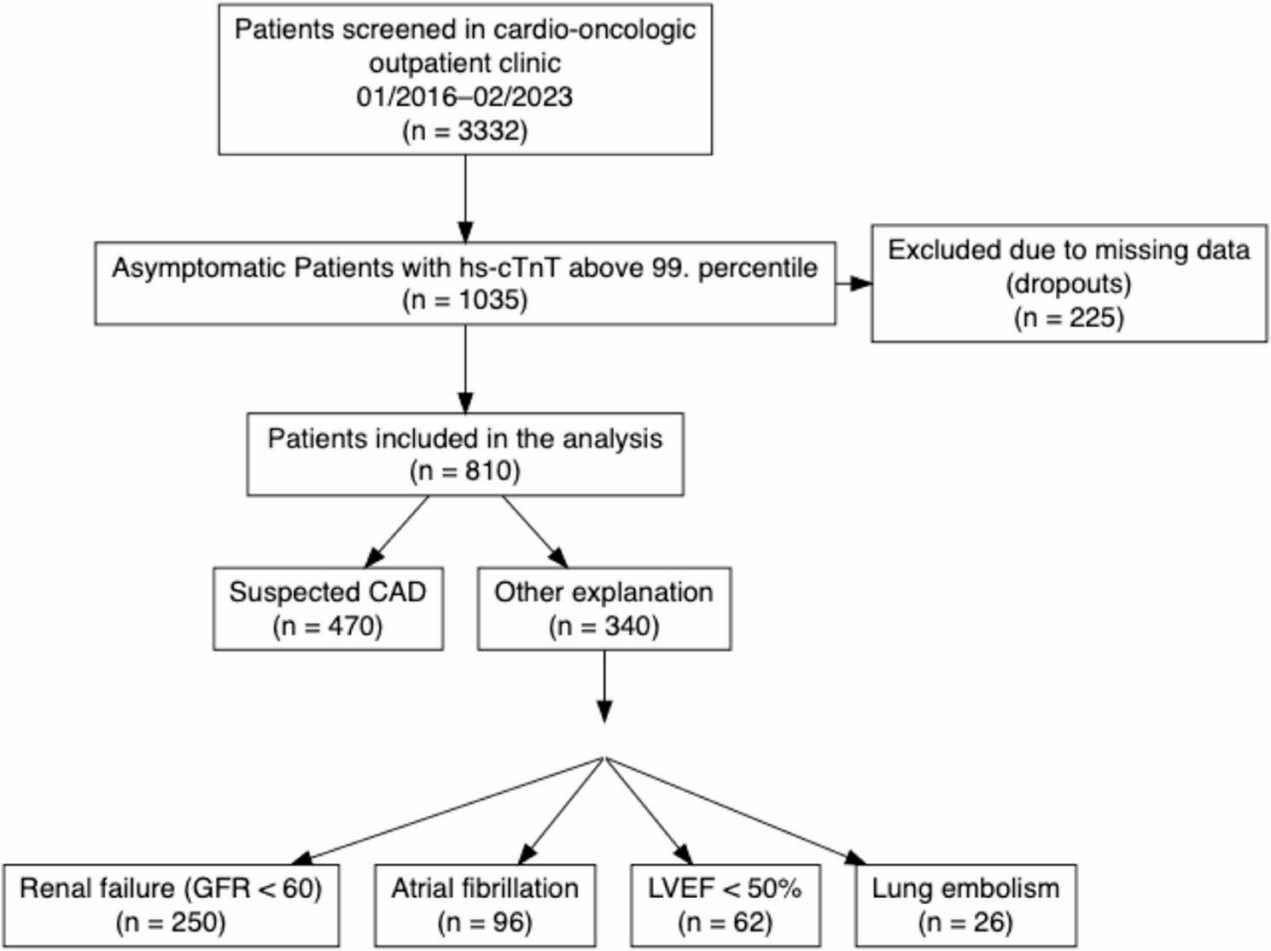
Patient flow chart. From January 2016 to February 2023, 3332 patients were screened in the cardio-oncology outpatient clinic. Among 1035 asymptomatic patients with hs-cTnT above the 99th percentile, 225 were excluded because of missing data. Among the remaining patients, 470 were classified as having suspected CAD, and 340 had alternative explanations. Abbreviations: CAD = coronary artery disease; GFR = glomerular filtration rate; hs-cTnT = high-sensitivity cardiac troponin T; LVEF = left ventricular ejection fraction

Between January 2016 and February 2023, a total of 3,332 patients were screened in our cardio-oncology outpatient clinic at Heidelberg University Hospital. Among these patients, 1,035 (31.1%) asymptomatic patients had high-sensitivity cardiac troponin T (hs-cTnT) levels above the 99th percentile upper limit of normal (ULN). After the exclusion of 225 patients due to incomplete data, 810 patients remained for further analysis. Within this cohort, 470 of 810 patients (58.0%) underwent further evaluation for suspected coronary artery disease (CAD), whereas in 340 of 810 patients (42.0%), an alternative explanation for troponin elevation was identified. The most frequent non-ischaemic causes were CKD (*n* = 250, 30.9%), atrial fibrillation (*n* = 96, 11.9%), left ventricular systolic dysfunction with LVEF < 50% (*n* = 62, 7.7%), and pulmonary embolism (*n* = 26, 3.2%) **(**Fig. 1**)**. Most patients with left ventricular systolic dysfunction exhibited stable left ventricular function, and new or worsening systolic impairment was uncommon (Fig. 10 in Appendix). In cases with reduced EF, underlying diagnoses were largely non-ischemic, and the majority were already receiving guideline-directed heart failure therapy (Fig. 10 in Appendix).

**Fig. 2:**
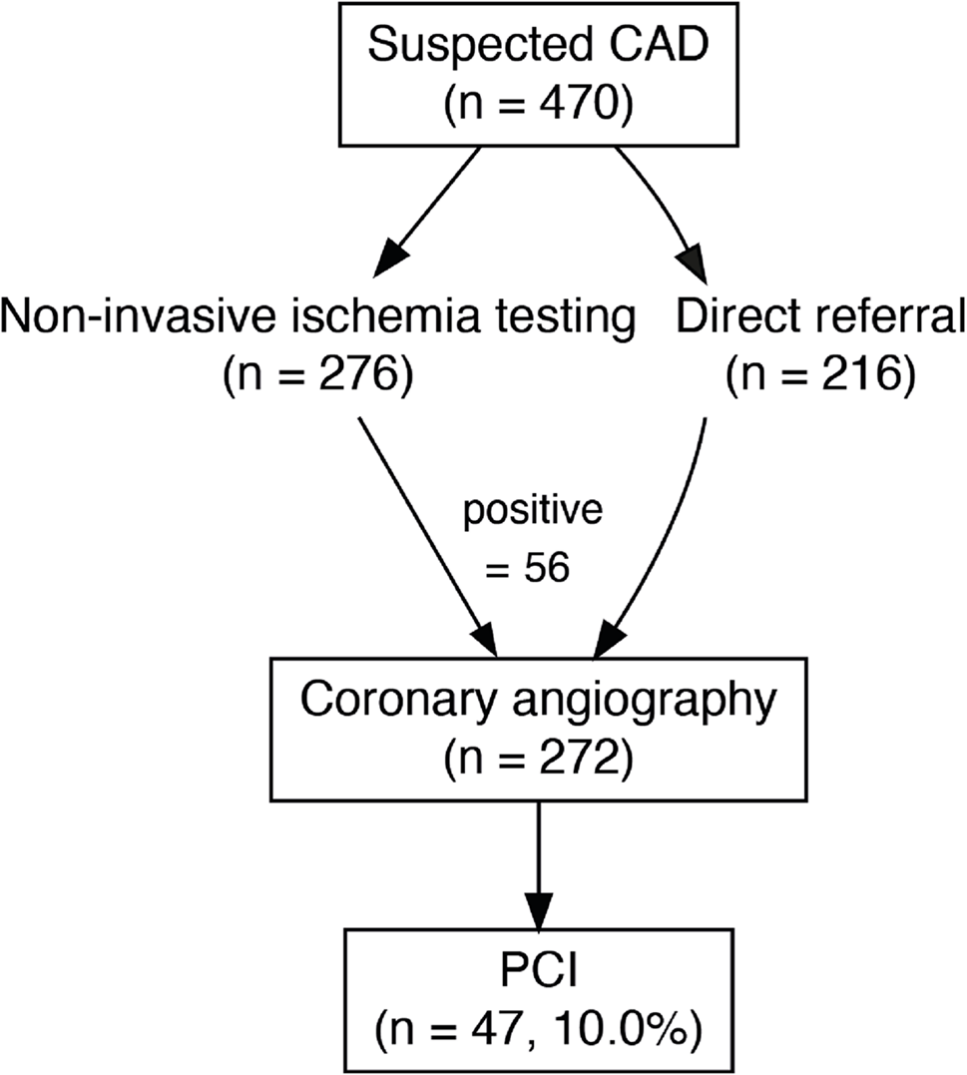
Diagnostic pathway of asymptomatic patients with elevated hs-cTnT. In the suspected CAD group, 276 patients underwent non-invasive ischemia testing, of which 56 had positive results. In total, 273 patients underwent coronary angiography, and 47 patients (10.0%) received PCI. Abbreviations: CAD = coronary artery disease; hs-cTnT = high-sensitivity cardiac troponin T; PCI = percutaneous coronary intervention

Among the 470 patients with suspected CAD, 276 (58.7%) underwent non-invasive ischemia testing, of whom 56 demonstrated evidence of myocardial ischemia (Fig. 2). In total, 273 of the 470 patients (58.1%) underwent invasive coronary angiography. Obstructive CAD was confirmed in 47 of the 273 cases, and percutaneous coronary intervention (PCI) was performed, corresponding to 10.0% of the CAD subgroup. Independent predictors of PCI among patients with elevated hs-cTnT are shown in Fig. 7 in Appendix.

For further analysis, the study cohort was stratified into patients with CAD (either new or known) and those without CAD (*n* = 404, 49.9%) (Table 1). Patients without CAD (*n* = 404, 49.9%) were younger on average (62.5 ± 15.1 vs. 69.9 ± 9.3 years, *p* < 0.001) and presented a more favourable cardiovascular risk profile. Compared with patients with CAD, those without CAD had lower rates of arterial hypertension (53.0% vs. 79.1%, *p* < 0.001), diabetes mellitus (13.6% vs. 25.6%, *p* < 0.001), and smoking (40.8% vs. 48.8%, *p* = 0.028). Biomarker levels were also lower in the no-CAD group, with reduced hs-cTnT levels (23.6 ± 22.1 vs. 43.9 ± 80.5 pg/ml, *p* < 0.001) and NT-proBNP levels (1,362 vs. 2,972 pg/mL, *p* = 0.003). Echocardiography revealed a greater mean left ventricular ejection fraction in patients without CAD (55.9% vs. 50.4%, *p* < 0.001). Lipid parameters, including total cholesterol and HDL cholesterol, did not differ between the two groups.

**Table 1:** Baseline characteristics of the study cohort

	Overall	No CAD	CAD	*p*
*n*	810	404	406	
Age, years (mean (SD))	66.18 (13.08)	62.45 (15.14)	69.88 (9.27)	< 0.001
Weight, kg (mean (SD))	78.12 (17.08)	76.67 (17.32)	79.53 (16.75)	0.018
BMI, kg/m2 (mean (SD))	26.44 (5.11)	26.22 (5.36)	26.66 (4.85)	0.231
Height, cm (mean (SD))	171.60 (9.39)	170.86 (10.09)	172.33 (8.60)	0.027
Cardiac Risk Factors				
Art. Hypertension (%)	535 (66.0)	214 (53.0)	321 (79.1)	< 0.001
Diabetes (%)	159 (19.6)	55 (13.6)	104 (25.6)	< 0.001
Smoking (%)	363 (44.8)	165 (40.8)	198 (48.8)	0.028
History of CAD (%)	216 (26.7)	0 (0.0)	216 (53.2)	< 0.001
eGFR (CKD-EPI), mL/min/1.73 m²	75.66 (26.11)	80.41 (27.93)	70.92 (23.25)	< 0.001
Biomarkers				
Total cholesterol, mg/dl (mean (SD))	180.86 (48.12)	184.29 (46.91)	177.82 (49.07)	0.182
HDL, mg/dl (mean (SD))	51.97 (17.76)	53.49 (19.82)	50.66 (15.71)	0.126
NT-proBNP, pg/mL (mean (SD))	2174.50 (7551.2)	1362.42 (4539.3)	2972.25 (9573.3)	0.003
Hs-cTnT, pg/mL (mean (SD))	33.75 (59.87)	23.62 (22.13)	43.89 (80.51)	< 0.001
Alternative causes for hs-cTnT elevation				
LVEF (mean (SD))	53.11 (10.36)	55.86 (8.18)	50.37 (11.52)	< 0.001
Pulmonary embolism (%)	60 (7.4)	29 (7.2)	31 (7.6)	0.909
Atrial fibrillation (%)	173 (21.4)	81 (20.0)	92 (22.7)	0.412

Baseline demographic, clinical, biomarker, and echocardiographic characteristics of the study cohort (*N* = 810), stratified by the presence or absence of coronary artery disease (CAD). Data are presented as the *n* (%) or mean ± SD, as appropriate. *P* values were calculated for comparisons between patients with and without CAD via the chi-square test or Fisher’s exact test for categorical variables and Student’s t test for continuous variables

*Abbreviations*: *BMI* body mass index, *CAD* coronary artery disease, *GFR* glomerular filtration rate, *HDL* high-density lipoprotein, *hs-cTnT* high-sensitivity cardiac troponin T, *LVEF* left ventricular ejection fraction, *NT-proBNP* N-terminal pro–B-type natriuretic peptide, *SD* standard deviation

### Distribution of tumour entities and CAD association

**Fig. 3:**
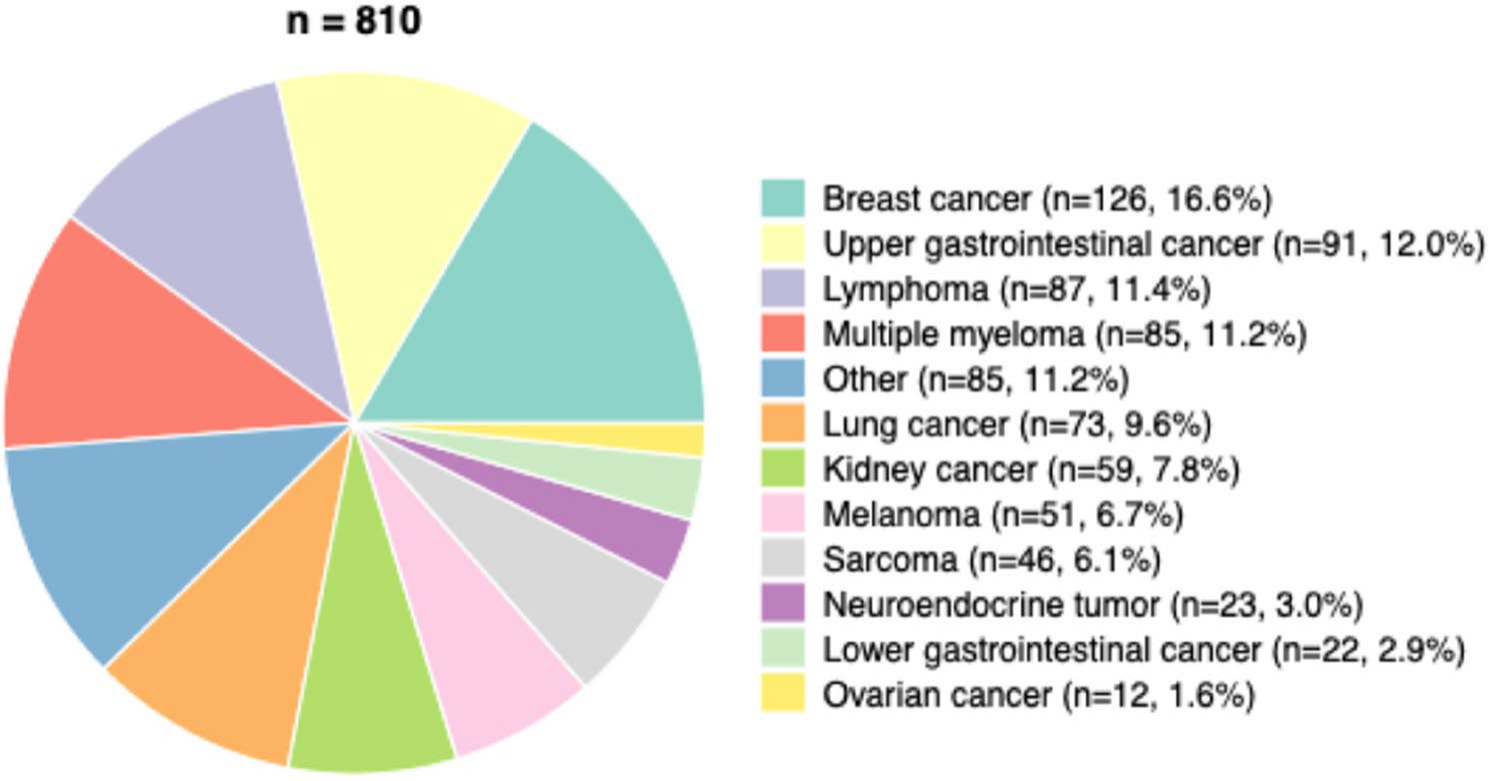
Distribution of Cancer Types in the Study Cohort. Cancer types among the study population (*n* = 810). Abbreviations: NET = neuroendocrine tumour

Among the 810 patients included in the final analysis, a broad spectrum of tumour entities was represented (Fig. 3). The most common malignancy was breast cancer (*n* = 126, 16.6%), followed by upper gastrointestinal cancer (*n* = 91, 12.0%), lymphoma (*n* = 87, 11.4%), and multiple myeloma (*n* = 85, 11.2%). Less frequent entities included lung cancer (*n* = 73, 9.6%), kidney cancer (*n* = 59, 7.8%), melanoma (*n* = 51, 6.7%), sarcoma (*n* = 46, 6.1%), neuroendocrine tumours (*n* = 23, 3.0%), lower gastrointestinal cancer (*n* = 22, 2.9%), and ovarian cancer (*n* = 12, 1.6%).

When analysing tumour entities according to CAD status (Table 2), breast cancer was significantly more common among patients without CAD (24.5% vs. 6.7%, *p* < 0.001), whereas lung cancer (13.1% vs. 5.0%, *p* < 0.001) and multiple myeloma (14.8% vs. 6.2%, *p* < 0.001) were more prevalent in the CAD group. Sarcoma occurred more frequently in patients without CAD (7.4% vs. 3.9%, *p* = 0.046). Other tumour types, including upper gastrointestinal, lower gastrointestinal, kidney, leukaemia, neuroendocrine, and ovarian cancers, and the heterogeneous “other” category, were not significantly different between the groups.

**Table 2:** Cancer types and timing of hs-cTnT elevation

	Overall	No CAD	CAD	*p*
*n*	810	404	406	
Upper gastrointestinal malignancies, n (%)	91 (11.2)	40 (9.9)	51 (12.6)	0.277
Multiple myeloma, n (%)	85 (10.5)	25 (6.2)	60 (14.8)	< 0.001
Lower gastrointestinal malignancies, n (%)	22 (2.7)	10 (2.5)	12 (3.0)	0.838
Breast cancer, n (%)	126 (15.6)	99 (24.5)	27 (6.7)	< 0.001
Lung cancer, n (%)	73 (9.0)	20 (5.0)	53 (13.1)	< 0.001
Kidney cancer, n (%)	59 (7.3)	25 (6.2)	34 (8.4)	0.288
Leukaemia, n (%)	50 (6.2)	23 (5.7)	27 (6.7)	0.675
Sarcoma, n (%)	46 (5.7)	30 (7.4)	16 (3.9)	0.046
NET, n (%)	23 (2.8)	9 (2.2)	14 (3.4)	0.404
Ovarian cancer, n (%)	12 (1.5)	9 (2.2)	3 (0.7)	0.144
Other, n (%)	85 (10.5)	45 (11.1)	40 (9.9)	0.629
Timing of hs-cTnT Elevation				
Before oncological therapy, n (%)	324 (40.0)	182 (45.0)	142 (35.1)	0.005
After oncological therapy, n (%)	135 (16.7)	66 (16.3)	69 (17.0)	0.863
During oncological therapy, n (%)	337 (41.7)	154 (38.1)	183 (45.2)	0.049

Distribution of underlying cancer types and timing of hs-cTnT elevation in the study cohort (*N* = 810), stratified by the presence or absence of coronary artery disease (CAD). The data are presented as *n* (%) as appropriate. *P* values were calculated for comparisons between patients with and without CAD via the chi-square test or Fisher’s exact test.

*Abbreviations*: *CAD* coronary artery disease, *hs-cTnT* high-sensitivity cardiac troponin T, *NET* neuroendocrine tumor

Furthermore, the timing of hs-cTnT elevation also differed between the groups. Elevation prior to the initiation of oncologic therapy was more frequently observed in the no-CAD group (45.0% vs. 35.1%, *p* = 0.005), whereas troponin elevation during therapy was more frequent in patients with CAD (45.2% vs. 38.1%, *p* = 0.049). Posttherapy elevations occurred at similar rates between the groups (17.0% vs. 16.3%, *p* = 0.863).

### Predictors of CAD-independent troponin elevation

**Fig. 4:**
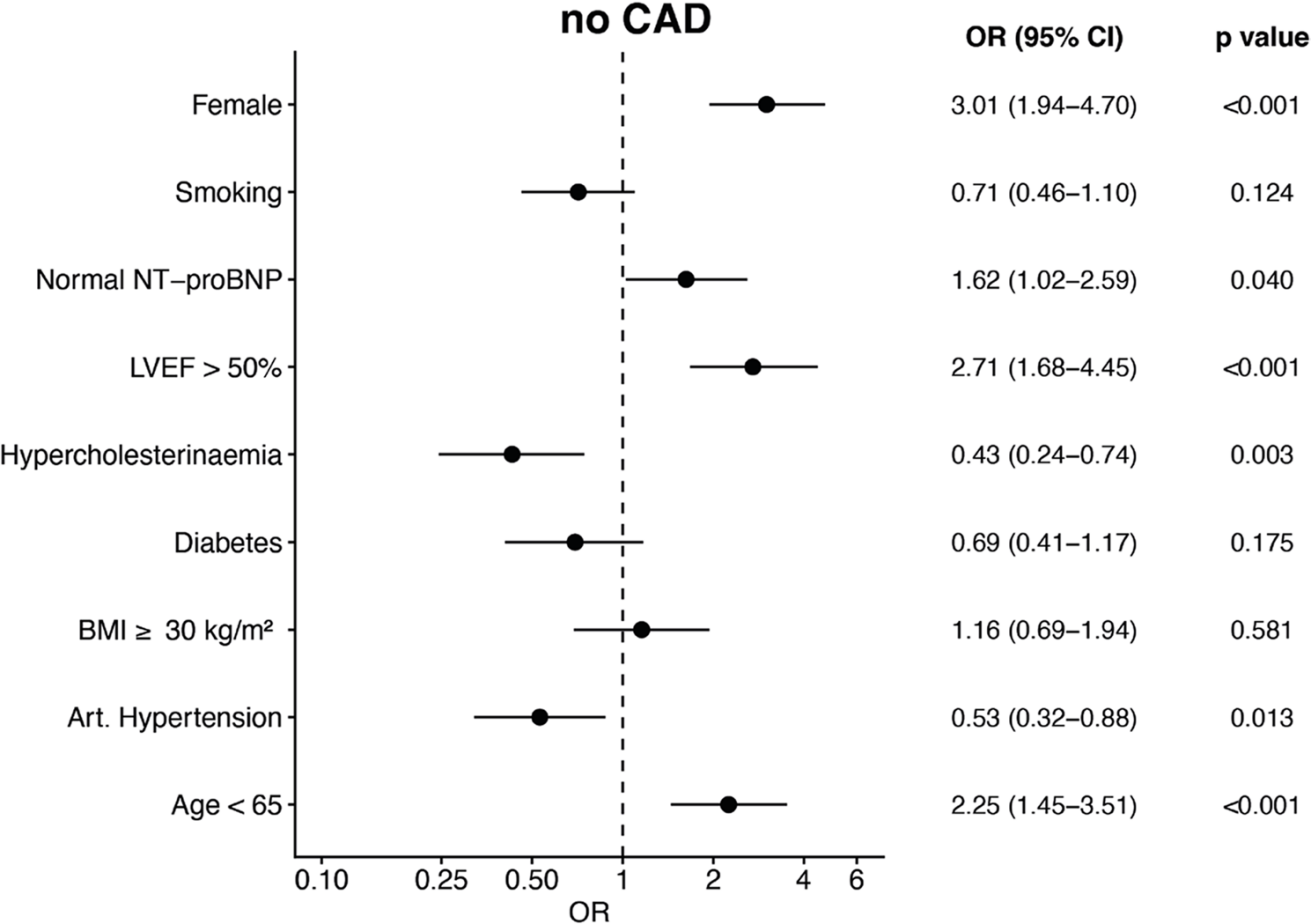
Predictors of CAD-independent increased troponin in asymptomatic cancer patients. Multivariate logistic regression analysis identifying independent predictors of the absence of coronary artery disease (CAD) among asymptomatic patients with elevated hs-cTnT. Odds ratios (ORs) with 95% confidence intervals (CIs) are shown on a logarithmic scale. Abbreviations: BMI = body mass index; CAD = coronary artery disease; hs-cTnT = high-sensitivity cardiac troponin T; LVEF = left ventricular ejection fraction; NT-proBNP = N-terminal pro–B-type natriuretic peptide; OR = odds ratio

Multivariate logistic regression identified several independent predictors for the absence of CAD (Fig. 4). Female sex (OR 3.01, 95% CI 1.94–4.70; *p* < 0.001), younger age (< 65 years; OR 2.25, 95% CI 1.45–3.51; *p* < 0.001), preserved LVEF ≥ 50% (OR 2.71, 95% CI 1.68–4.45; *p* < 0.001), and normal levels of NT-proBNP adjusted for age (OR 1.62, 95% CI 1.02–2.59; *p* = 0.040) were strong positive predictors of the absence of CAD.

In contrast, arterial hypertension (OR 0.53, 95% CI 0.32–0.88; *p* = 0.013) and hypercholesterinaemia (OR 0.43, 95% CI 0.24–0.74; *p* = 0.003) were inversely associated with the absence of CAD. Diabetes mellitus, obesity (BMI ≥ 30 kg/m²), and smoking were not significantly associated with CAD after adjustment. Multivariable models for the presence of CAD are provided in Fig. 8 in Appendix.

### Troponin concentrations across chemotherapy time points

**Fig. 5:**
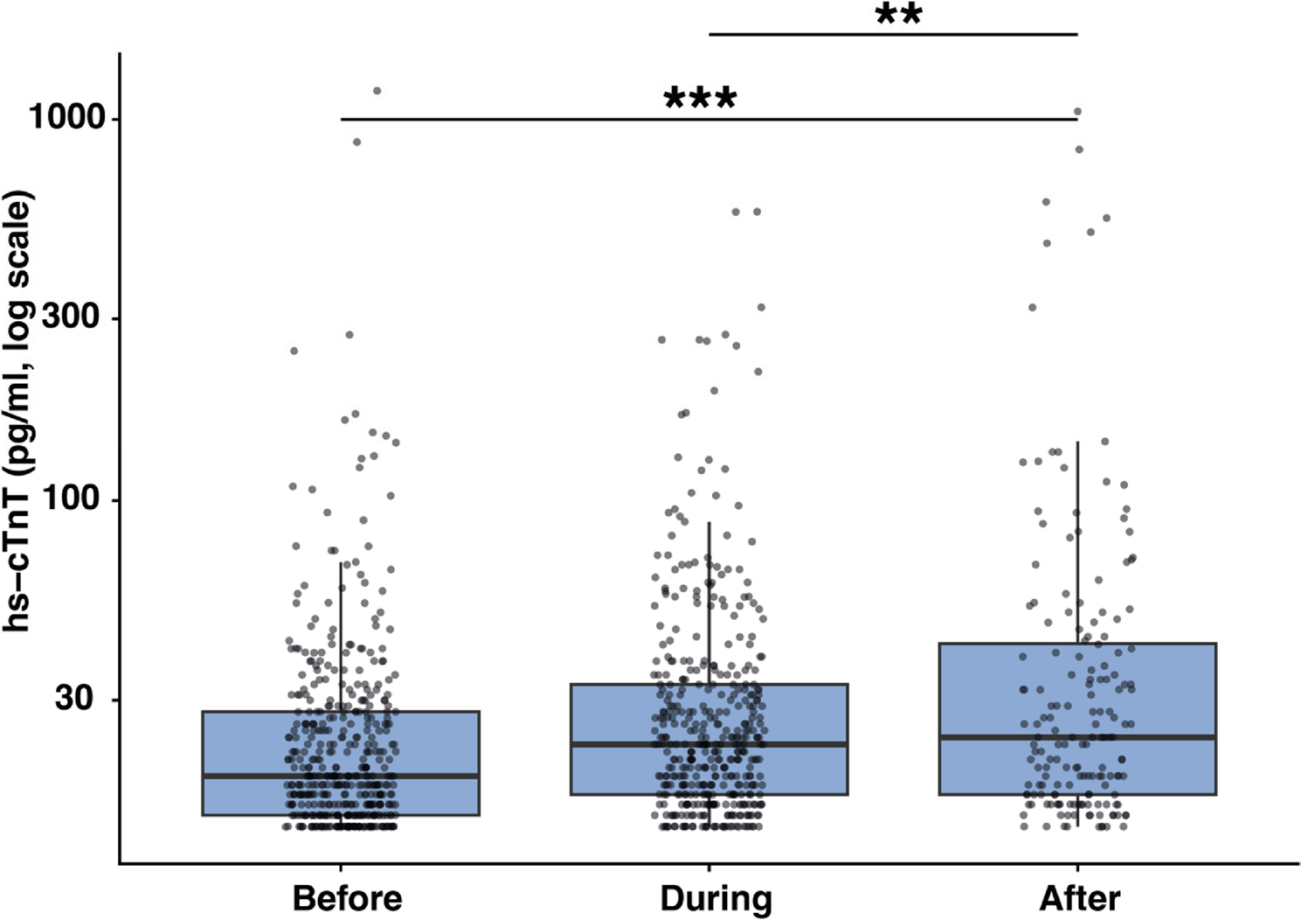
Distribution of troponin levels of all patients across chemotherapy time points. Box- and scatterplot illustrating the distribution of hs-cTnT concentrations measured before, during, and after chemotherapy administration. Troponin values are shown on a logarithmic scale. The central line indicates the median, boxes indicate the interquartile range, and whiskers indicate the 1.5× interquartile range. ANOVA with Bonferroni-adjusted post-hoc comparison, ** *p* < 0.01, *** *p* < 0.001

Troponin concentrations differed significantly across the chemotherapy time points (Fig. 5). Median troponin concentrations were lowest before treatment, slightly higher during ongoing chemotherapy, and highest after chemotherapy completion. Post-hoc comparisons showed significantly elevated hs-cTnT values after chemotherapy compared with both the before- and during-treatment periods.

### Patterns of oncological treatment in asymptomatic cancer patients with elevated hs-cTnT

**Fig. 6:**
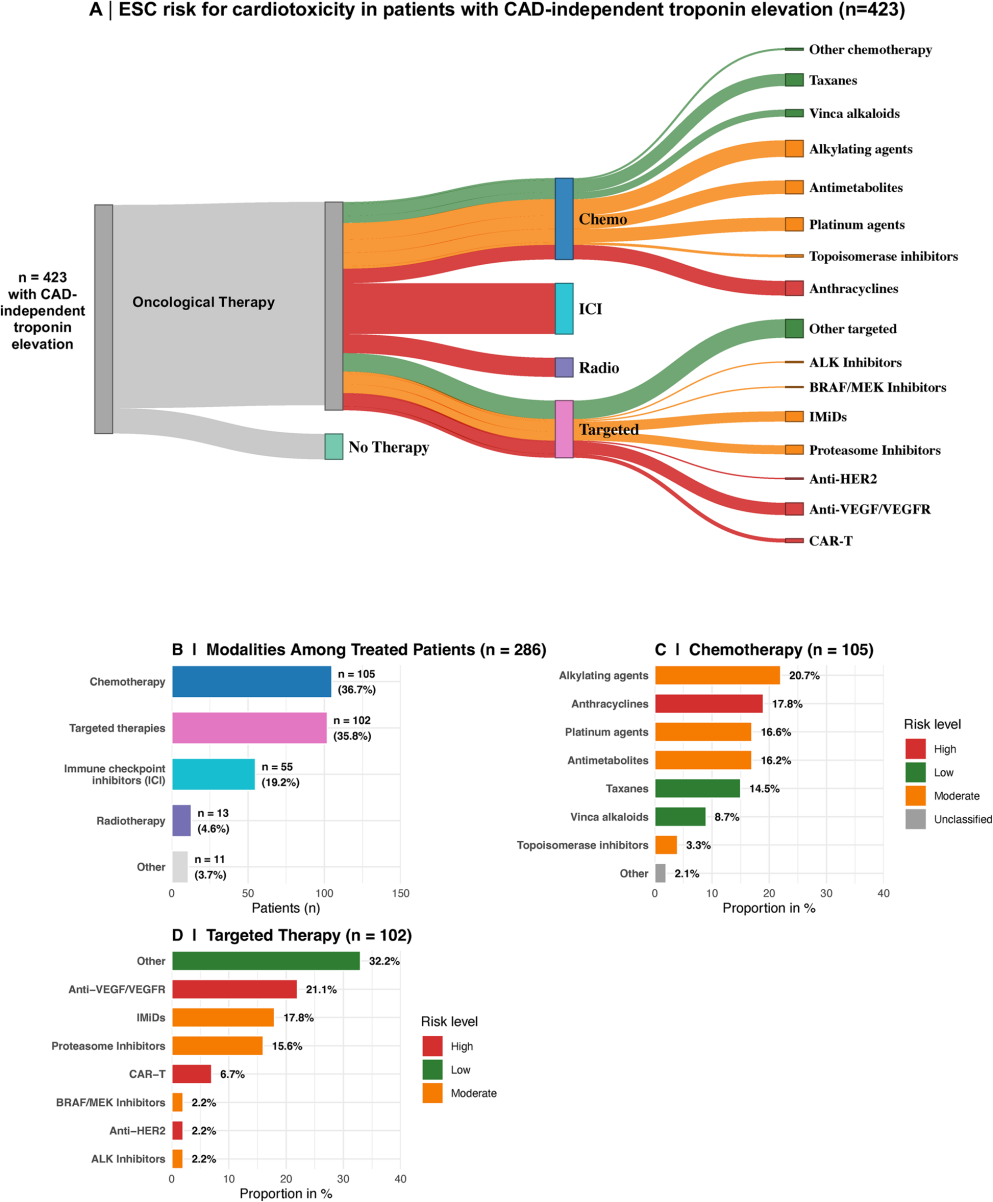
Distribution of cancer therapy modalities and associated cardiotoxicity risk profiles. **A** Sankey plot illustrating the distribution of patients according to treatment modality: chemotherapy (Chemo), immune checkpoint inhibitors (ICIs), radiotherapy (Radio), targeted therapies, or no oncological therapy. Subtypes of chemotherapy and targeted agents are further specified. **B** Overall distribution of treatment modalities among treated patients (*n* = 286). **C** Subtypes of chemotherapy (*n* = 105) with corresponding cardiotoxicity risk classifications according to the ESC Cardio-oncology guidelines. **D** Subtypes of targeted therapy (*n* = 102) with corresponding risk classifications. Risk levels were categorized as high (red), moderate (orange), or low (green). Abbreviations: ALK = anaplastic lymphoma kinase; BRAF = v-Raf murine sarcoma viral oncogene homologue B; CAR-T = chimeric antigen receptor T cell; HER2 = human epidermal growth factor receptor 2; ICI = immune checkpoint inhibitor; IMiDs = immunomodulatory drugs; MEK = mitogen-activated protein kinase; Radio = radiotherapy; VEGF/VEGFR = vascular endothelial growth factor/receptor

**Fig. 7:**
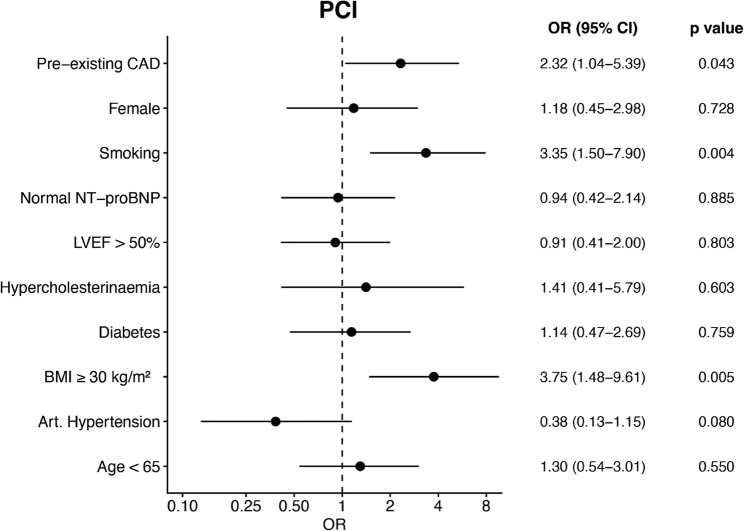
Predictors for PCI in asymptomatic cancer patients. Multivariate logistic regression analysis identifying independent predictors of PCI among asymptomatic patients with elevated hs-cTnT. Odds ratios (ORs) with 95% confidence intervals (CIs) are shown on a logarithmic scale. Abbreviations: BMI = body mass index; CAD = coronary artery disease; hs-cTnT = high-sensitivity cardiac troponin T; LVEF = left ventricular ejection fraction; NT-proBNP = N-terminal pro–B-type natriuretic peptide; OR = odds ratio

**Fig. 8:**
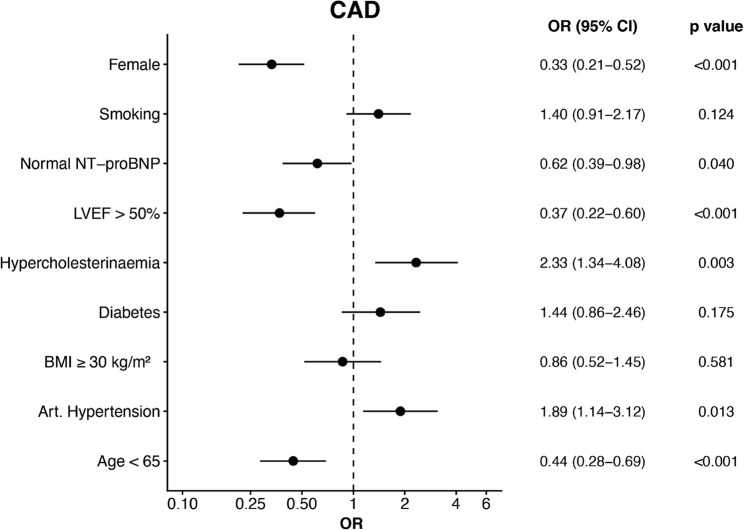
Predictors of CAD in asymptomatic cancer patients. Multivariate logistic regression analysis identifying independent predictors of CAD among asymptomatic patients with elevated hs-cTnT. Odds ratios (ORs) with 95% confidence intervals (CIs) are shown on a logarithmic scale. Abbreviations: BMI = body mass index; CAD = coronary artery disease; hs-cTnT = high-sensitivity cardiac troponin T; LVEF = left ventricular ejection fraction; NT-proBNP = N-terminal pro–B-type natriuretic peptide; OR = odds ratio

**Fig. 9:**
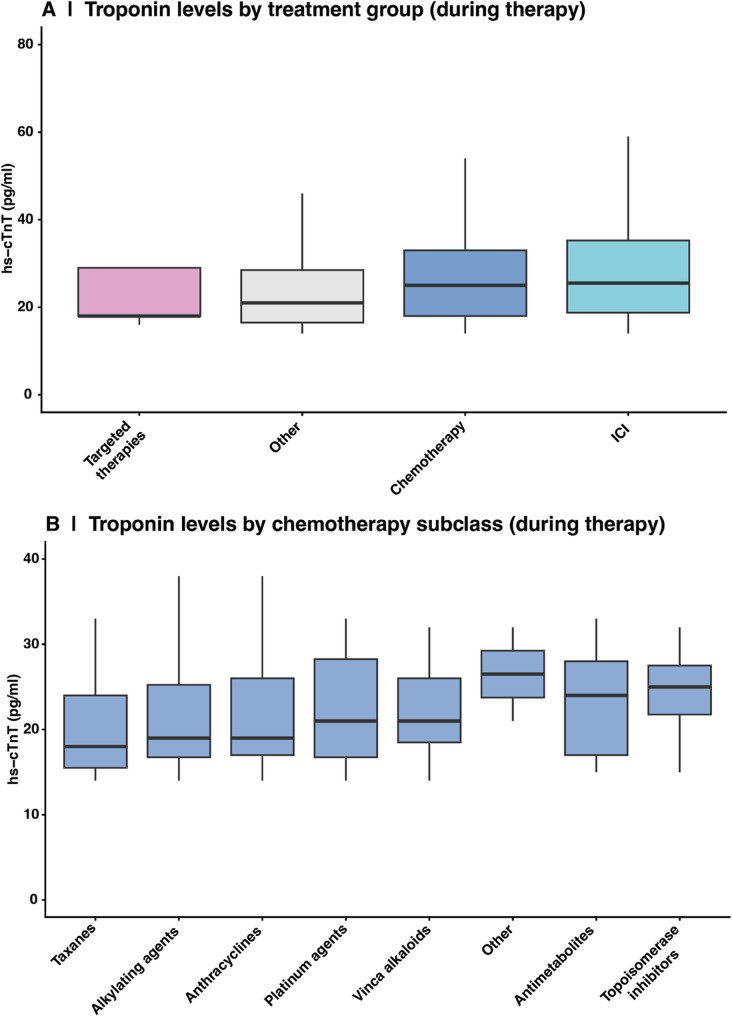
Troponin levels by treatment group (**A**) and chemotherapy subclass (**B**) during therapy. Boxplots depicting high-sensitivity cardiac troponin T (hs-cTnT) concentrations (pg/ml) stratified by major treatment groups (**A**) and chemotherapy subclasses (**B**). The central line indicates the median, boxes indicate the interquartile range, and whiskers indicate the 1.5× interquartile range

**Fig. 10:**
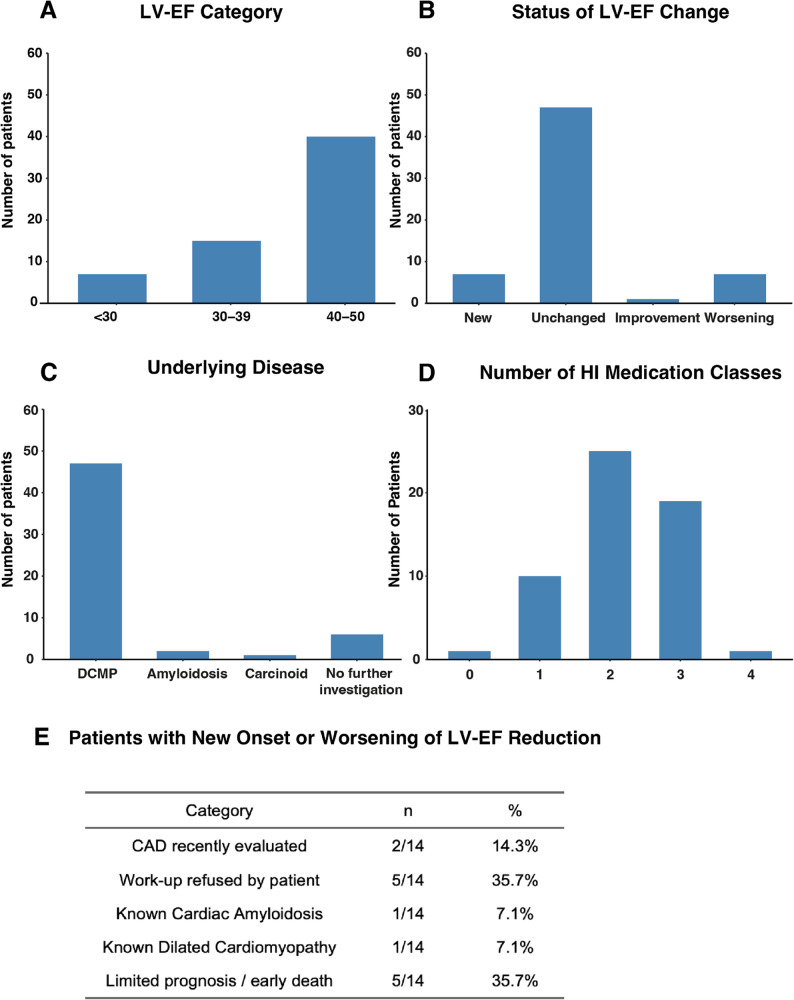
Left ventricular ejection fraction (LVEF) characteristics and underlying aetiologies. **A** Distribution of LVEF categories among patients with reduced systolic function. **B** Status of LVEF change compared with previous imaging. **C** Underlying cardiac conditions associated with reduced LVEF. **D** Number of heart failure medication classes prescribed per patient. **E** Clinical characteristics of patients with new-onset or worsening LVEF

47 of 470 (10.0%) patients evaluated for suspected CAD underwent PCI due to obstructive CAD. The remaining 423 of 470 patients (90.0%) presented elevated hs-cTnT levels without the need for myocardial revascularization. Among these patients, two-thirds (*n* = 286, 67.6%) received oncologic therapy, whereas one-third (*n* = 137, 32.4%) had not received active cancer treatment (Fig. 6). Within the treated subgroup, the most frequent modalities were targeted therapies (35.8%) and chemotherapy (36.7%), followed by immune checkpoint inhibitors (19.2%), radiotherapy (4.6%), and other therapies (3.7%) (Fig. 6). The chemotherapy regimens used were heterogeneous (Fig. 6). Detailed distributions of hs-cTnT concentrations by treatment group and chemotherapy subclass are shown in Fig. 9 in Appendix. The most common agents were alkylating agents (20.7%), anthracyclines (17.8%), platinum compounds (16.6%), and antimetabolites (16.2%), followed by taxanes (14.5%), vinca alkaloids (8.7%), and topoisomerase inhibitors (3.3%). Other types of chemotherapy accounted for 2.1% of the patients.

Oncologic therapies administered in this cohort span the full spectrum of cardiotoxic risk according to the 2022 ESC cardio-oncology guidelines. Among the 286 patients who received active treatment, approximately one-third (34.3%) were exposed to high-risk agents, most commonly anthracyclines, immune checkpoint inhibitors, CAR-T-cell therapy, anti-VEGF/VEGFR inhibitors, anti-HER2 therapy, or radiotherapy. Nearly half (44.0%) of the patients received moderate-risk therapies, including alkylating agents, platinum compounds, antimetabolites, proteasome inhibitors, IMiDs, ALK inhibitors, BRAF/MEK inhibitors, and topoisomerase inhibitors. The remaining 21.7% were treated exclusively with low-risk drugs, such as taxanes, vinca alkaloids, or other chemotherapy/targeted agents, without established cardiotoxicity. Overall, more than three quarters of treated patients (78.3%) were exposed to at least one moderate- or high-risk cardiotoxic therapy.

## Discussion

In this large cohort of asymptomatic oncologic patients with unexplained elevated hs-cTnT, obstructive CAD is responsible for only a minority of cases, whereas most biomarker elevations are related to cancer or cancer therapy. A total of 78% of actively treated patients without obstructive CAD were exposed to at least one oncologic therapy categorized as moderate- or high-risk for cardiotoxicity according to ESC guidelines, which may reflect the suspected cause of biomarker elevation [24].

**Fig. 11:**
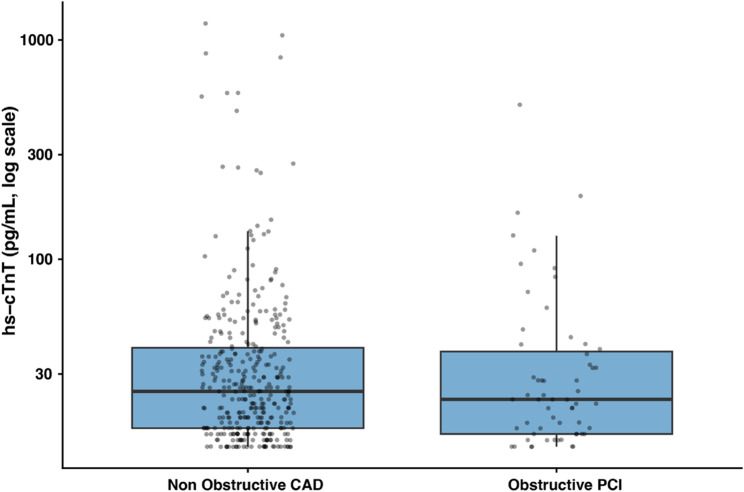
Troponin levels in patients with non-obstructive CAD versus obstructive CAD requiring PCI. Distribution of high-sensitivity cardiac troponin T (hs-cTnT) concentrations in patients with non-obstructive coronary artery disease and those with obstructive CAD requiring percutaneous coronary intervention (PCI). Values are displayed on a logarithmic scale. Boxplots indicate the median and interquartile range. Group differences were assessed using the unpaired Student’s t test

While hs-cTnT is an established marker of myocardial injury, its diagnostic specificity for CAD is challenging in oncology, where concomitant factors such as renal dysfunction, atrial fibrillation, pulmonary embolism, and direct treatment-related toxicity are common [25–28]. We observed that nearly 90% of patients with elevated hs-cTnT and suspected CAD ultimately showed no indication for revascularization, supporting the concept that troponin elevation in this setting should not be equated with obstructive ischemic heart disease [26, 29, 30]. In chronic LV dysfunction, mild hs-cTnT elevations typically reflect myocardial stress and remodelling processes rather than ischaemia, which is consistent with the largely non-ischaemic causes observed in our study. However, elevated hs-cTnT is a strong indicator of increased risk for cardiotoxicity and increased all-cause mortality [31]. In patients with known CAD, hs-cTnT levels did not clearly discriminate between non-obstructive coronary disease and obstructive lesions requiring PCI (Fig. 11 in Appendix).

**Fig. 12:**
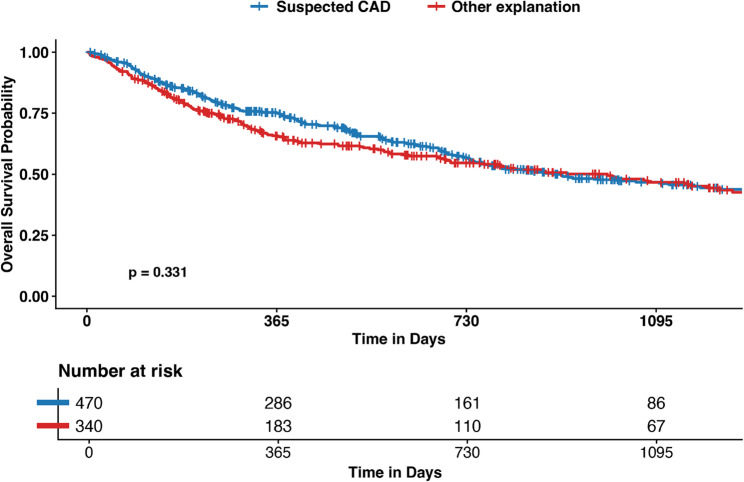
Overall survival in patients with suspected CAD versus an alternative explanation for hs-cTnT elevation.Kaplan–Meier curves comparing overall survival between patients who underwent diagnostic evaluation for suspected coronary artery disease (CAD) and those in whom a non-ischaemic explanation for hs-cTnT elevation was identified and no CAD work-up was pursued. Group differences were assessed using the log-rank test. Numbers at risk at each time point are displayed below the plot

Nevertheless, classical cardiovascular risk factors remain key drivers of coronary artery disease in cancer patients. Younger age, female sex, preserved left ventricular function, and normal NT-proBNP levels were independently associated with the absence of CAD, whereas other factors, such as arterial hypertension and hypercholesterinemia, were associated with the occurrence of CAD. Interestingly, troponin elevation during the course of oncologic therapy was more frequently detected in patients with CAD than in those without CAD. This finding may suggest a synergistic interaction between underlying coronary artery disease and cancer therapy–related cardiotoxicity, whereby preexisting CAD increases myocardial vulnerability to additional stressors such as chemotherapy or radiotherapy [16]. These findings indicate that although troponin elevations in cancer patients are often multifactorial, classical cardiovascular risk profiles remain essential for identifying those at risk of coronary disease. An important aspect of our cohort is the subgroup of 340 patients in whom further ischemic testing was not performed because a non-ischemic explanation for troponin elevation was present. To address potential concerns regarding the safety of this approach, we compared long-term survival with patients who underwent CAD evaluation (Fig. 12 in Appendix). Overall survival was similar in both groups suggesting that withholding further CAD work-up in clinically appropriate cases did not adversely affect patient outcomes. This supports current practice, in which invasive evaluation is guided by the overall clinical context rather than biomarker elevation alone.

**Fig. 13:**
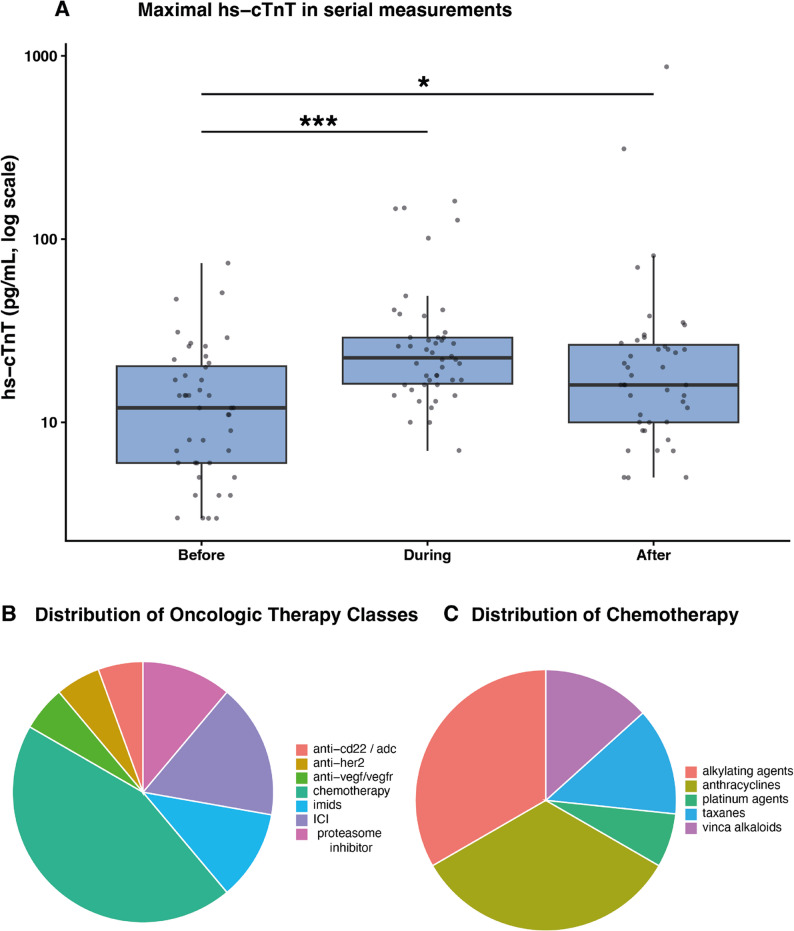
Maximal hs-cTnT in patients with measurements before, during, and after Therapy. **A** Maximal hs-cTnT concentrations before, during, and after anticancer therapy in patients with available measurements at all three time points. Boxplots show median and interquartile ranges on a logarithmic scale. ANOVA with Bonferroni-adjusted post-hoc comparison.*p < 0.05, ***p < 0.001. **B** Distribution of oncologic therapy classes among patients with serial troponin measurement. **C** Distribution of chemotherapy subclasses in this subgroup

**Fig. 14:**
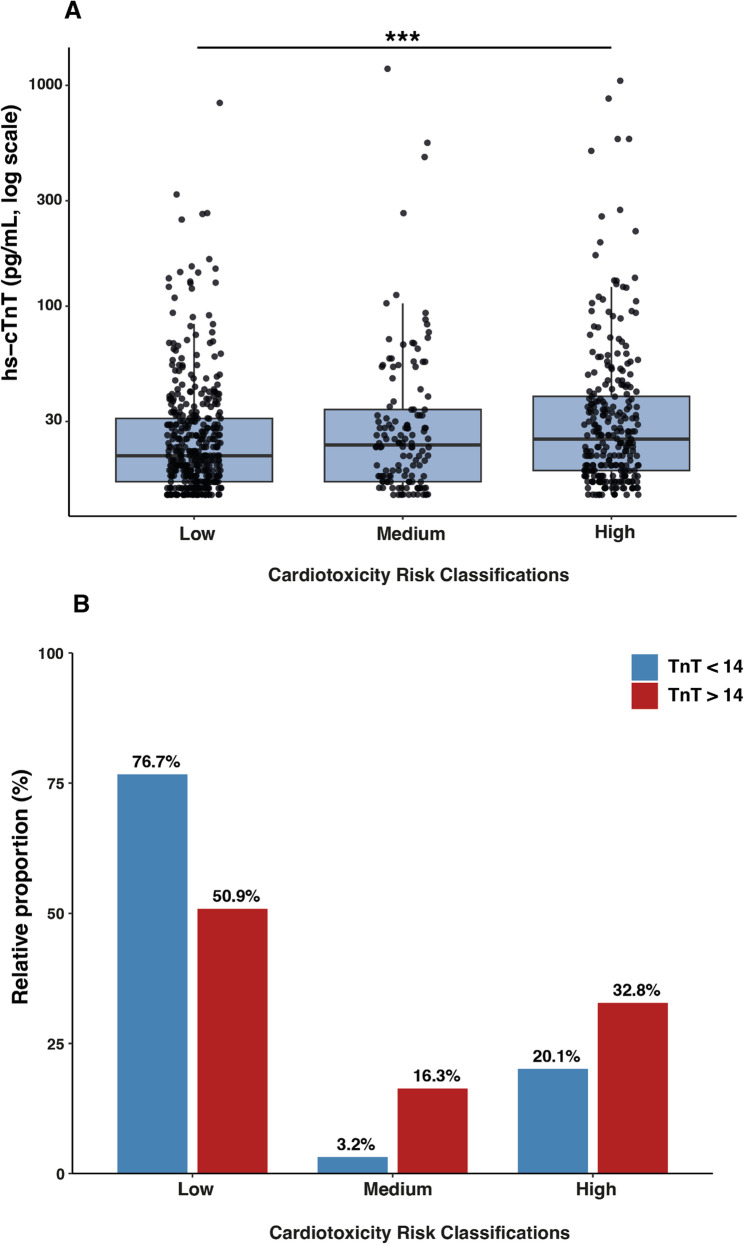
Hs-cTnT levels across cardiotoxicity risk categories. **A** Boxplots show median and interquartile ranges on a logarithmic scale in patients with elevated hs-cTnT classified as low, medium, or high cardiotoxicity risk based on their oncologic therapies. ANOVA with Bonferroni-adjusted post hoc comparison. ***p < 0.001. **B** Bar charts show the proportion of patients assigned to low, medium, or high cardiotoxicity risk categories, stratified by troponin status (hs-cTnT < 14 pg/mL (blue) vs. hs-cTnT > 14 pg/mL (red)). Percentages are displayed above each bar. Risk categories are based on ESC cardiotoxicity classifications derived from prior oncologic therapies

Oncologic therapy emerged as a major determinant of the risk for troponin elevation in this cohort. Troponin concentrations differed significantly across the chemotherapy timing. Median hs-cTnT values were lowest before treatment, increased slightly during ongoing chemotherapy, and reached their highest levels after completion of therapy. This supports earlier reports of cancer-therapy-induced cardiac damage based on hs-cTnT elevation [32]. To further substantiate this temporal pattern, we additionally analysed the subgroup of patients with serial hs-cTnT measurements available in all three phases. Maximal hs-cTnT values were lowest before therapy, increased during and after treatment (Fig. 13 in Appendix). These findings strengthen the concept of a progressive, treatment-related myocardial injury even in asymptomatic patients. Among the 286 patients with no evidence of obstructive CAD and who received active cancer treatment, > 75% were exposed to agents classified as having moderate or high cardiotoxic risk according to the 2022 ESC cardio-oncology guidelines [24]. To further examine the link between treatment-related cardiotoxicity and biomarker elevation, we analyzed hs-cTnT levels across ESC-defined cardiotoxicity risk categories. Median hs-cTnT values increased progressively from Low to High risk (Fig. 14 A in Appendix). Moreover, patients with hs-cTnT elevation (hs-cTnT > 14 pg/mL) were more frequently exposed to moderate- or high-risk therapies than troponin-negative patients (Fig. 14B in Appendix). Together, these findings support treatment-related myocardial stress as the predominant mechanism underlying asymptomatic hs-cTnT elevation.

Taken together, these findings underline two key implications for clinical practice. First, hs-cTnT elevation in cancer patients should not be automatically interpreted as evidence of obstructive CAD, and diagnostic strategies need to carefully balance ischaemic and non-ischaemic causes. Second, given the high prevalence of exposure to cardiotoxic agents, structured cardiovascular monitoring is essential, even in the absence of obstructive coronary disease. This approach is consistent with the 2022 ESC cardio-oncology guidelines, which emphasize systematic risk evaluation, biomarker follow-up, and multidisciplinary coordination [24].

Several limitations of this study should be acknowledged. First, this was a single-centre study, which may limit its generalisability. The cardiotoxicity risk classification was based on guideline categories and may not capture individual variability in susceptibility. Finally, long-term outcomes related to therapy-specific cardiotoxicity were not available. However, since we did not include patients with normal troponin levels, we cannot explore the incidence of troponin elevation among specific oncologic therapies. Nevertheless, the distribution of therapies among the analyzed patients did not point towards specific oncologic therapies that were more frequently associated with hs-cTnT elevation. Pre-test probability scores were not applied because all asymptomatic patients were classified as having a “very low” likelihood of obstructive CAD in these symptom-based models [33]. We therefore evaluated only patients without an alternative explanation for troponin elevation. However, this approach may introduce a bias that underestimates the true prevalence of obstructive CAD in this population. 

In conclusion, elevated hs-cTnT in cancer patients may more frequently reflect myocardial damage rather than obstructive CAD. Traditional cardiovascular risk factors are less predictive in this context, whereas oncologic therapy exposure plays a potential role. More than three-quarters of actively treated patients received moderate- or high-risk agents, highlighting the need for systematic cardio-oncology surveillance to mitigate therapy-related morbidity in this vulnerable population.

## Data Availability

The datasets generated and/or analysed during the current study are available from the corresponding author on reasonable request.
